# Viral Anxiety Mediates the Influence of Intolerance of Uncertainty on Adherence to Physical Distancing Among Healthcare Workers in COVID-19 Pandemic

**DOI:** 10.3389/fpsyt.2022.839656

**Published:** 2022-06-06

**Authors:** Seockhoon Chung, Taeyeop Lee, Youjin Hong, Oli Ahmed, Washington Allysson Dantas Silva, Jean-Philippe Gouin

**Affiliations:** ^1^Department of Psychiatry, Asan Medical Center, University of Ulsan College of Medicine, Seoul, South Korea; ^2^Department of Psychiatry, GangNeung Asan Hospital, University of Ulsan College of Medicine, Gangneung, South Korea; ^3^Department of Psychology, University of Chittagong, Chattogram, Bangladesh; ^4^National Centre for Epidemiology and Population Health, Australian National University, Canberra, ACT, Australia; ^5^Department of Psychology, Universidade Federal da Paraíba, João Pessoa, Brazil; ^6^Department of Psychology, Concordia University, Montreal, QC, Canada

**Keywords:** physical distancing, COVID-19, anxiety, uncertainty, stress

## Abstract

**Introduction:**

The aims of this study were to examine the mediation effect of viral anxiety of healthcare workers on the influence of their intolerance of uncertainty on the adherence to physical distancing during the COVID-19 pandemic.

**Methods:**

An online survey was conducted among 329 healthcare workers (female: 81.4%, nursing professionals: 59.0%, and shift workers: 22.3%) on November 29, 2021. Participants responded to questionnaires on adherence to physical distancing, health beliefs, and perceived social norms, and rating scales of the Stress and Anxiety to Viral Epidemics-6 items (SAVE-6), Patient Health Questionnaire-9 items (PHQ-9), and the Intolerance of Uncertainty-12 items (IUS-12) scale.

**Results:**

Adherence to physical distancing of healthcare workers was predicted by perceived benefits of physical distancing (β = 0.13, *p* = 0.01), personal injunctive norms (β = 0.32, *p* < 0.001), SAVE-6 score (β = 0.13, *p* = 0.02), and IUS-12 score (β = 0.10, *p* = 0.045) (adjusted R^2^ = 0.21, F = 22.3, *p* < 0.001). Viral anxiety mediated the association between intolerance of uncertainty and adherence to physical distancing but not the influence of perceived benefits and personal injunctive norms on adherence to physical distancing.

**Conclusion:**

We observed that viral anxiety of healthcare workers mediated the association between intolerance of uncertainty and adherence to physical distancing. During this pandemic, exploring adherence to physical distancing and its predicting factors will be helpful for the safety of healthcare workers and the patients for whom they care.

## Introduction

Since the onset of the COVID-19 pandemic in 2020 (1), people in all countries have suffered from distress related to the virus. As of March 28, 2022, there have been 481,213,782 confirmed COVID-19 cases and 6,150,003 recorded COVID deaths worldwide^1^, and 12,003,054 confirmed COVID-19 cases and 15,186 recorded COVID deaths in South Korea^2^. During the pandemic, frontline healthcare workers have suffered from psychological distress such as depression, anxiety, insomnia, fear of mortality, and post-traumatic stress disorder (2). Healthcare workers are facing the fear of infecting or transferring the virus to their family, friends, and colleagues, heavier workloads, perceived stigmatization, and increased scrutiny, and are coping by avoiding crowds and colleagues. To protect themselves and others, they must be fully vaccinated and follow the physical distancing guidelines (3); however, there were few reports on the adherence to physical distancing among healthcare workers (4).

### Psychological Distress of Physical Distancing

Physical distancing has been one of most effective measures for preventing transmission of the COVID-19 virus (5, 6). However, it has imposed large costs on society. In this context, the Korean government announced the living with COVID-19 (“living-with-corona”) policy and began to prepare residents for a return to the “new normal.” Although physical distancing is beneficial, it is also known to cause psychological distress. Social isolation has been associated with increased fear, anxiety symptoms, loneliness, and depressed mood (7), perhaps due to a long period of isolation or the economic burden it carries with it. Social isolation is different from social distancing; however, social distancing seems to be related to social isolation, therefore the term “physical distancing” is now used to reduce feelings of social isolation that are associated with the term “social distancing” (8). Adherence to physical distancing is important in disease prevention and control, despite the negative impact on psychological distress. Previous studies have shown that a sense of personal responsibility and control over one's own behavior is related with adherence to physical distancing (9). Familial support has also been reported to play an important role in improving adherence (10), while decreased psychosocial well-being and lack of social support were related to non-adherence (11).

### Viral Anxiety, Depression, Intolerance of Uncertainty, and Adherence to Physical Distancing

Throughout the COVID-19 pandemic, viral anxiety has been reported to be associated with adherence to or compliance with physical distancing. Anxiety may influence people's physical activity and time spent outdoors (12); thus, it has been reported that people who feel anxiety tend to adhere to physical distancing (13–15). “Sodisphobia,” or viral anxiety, is defined as experiencing excessive anxiety of being infected with viruses while in public (15). Although viral anxiety is thought to influence adherence to physical distancing, lower levels of anxiety and depression have also been reported to be associated with perceived compliance with physical distancing (16). Depression has been reported to be a predictor for physical distancing fears (17), while lower levels of depression have been reported to be associated with better adherence to measures of physical distancing (18). In general, high levels of depression have been associated with poor compliance to recommendations (19), and patients' depression is related to their non-adherence to medical treatment (20). Therefore, we can speculate that depressive symptoms of healthcare workers may be related to reduced adherence to physical distancing.

Intolerance of uncertainty, or the inability to successfully process and respond to information in uncertain contexts (21, 22), was reported to be associated with symptoms of anxiety (23, 24). Generally, intolerance of uncertainty is considered to be specific risk factor or cognitive vulnerability in the development and maintenance of anxiety disorders (25). Conceptually, intolerance of uncertainty is associated with generalized anxiety disorder (26) and obsessive-compulsive disorder (27). Difficulty tolerating uncertainty can manifest as cognitive and behavioral attempts to reduce uncertainty and enhance control (21). In the COVID-19 era, healthcare workers may find it difficult to tolerate the uncertainty associated with the spread of COVID-19. This may cause them to enhance their adherence to physical distancing to ensure the safety of their patients and themselves. Therefore, we can speculate that intolerance of uncertainty and viral anxiety may influence adherence to physical distancing. Depression also may be related with intolerance of uncertainty. Intolerance of uncertainty is associated with the etiology of depression (28). Further, it was reported that eliminating uncertainty from COVID-19 may reduce depressive symptoms among the general population (29). However, it is unclear whether healthcare workers' intolerance of uncertainty regarding COVID-19 contributes to depression or vice versa. Depression has previously been associated with decreased adherence to physical distancing, so it is essential to explore whether healthcare workers' intolerance of uncertainty influences depression to understand their level of adherence to physical distancing.

### Aims of the Study

In this study, we first aimed to explore the reliability and validity of the questionnaires on adherence to physical distancing and health beliefs model proposed by Gouin et al. (30) among healthcare workers. Most healthcare workers adhered to the physical distancing policy during this COVID-19 pandemic, a meaningful and valuable behavior for their own safety and the safety of their patients, although it caused them stress and emotional distress. Therefore, the validated Korean version of the scale will be useful to assess adherence of healthcare workers to physical distancing policy during the COVID-19 outbreak.

Second, we aimed to examine the relationships among adherence to physical distancing, viral anxiety, depression, and intolerance of uncertainty in healthcare workers during the COVID-19 pandemic. We hypothesized that intolerance of uncertainty of healthcare workers may be associated with their adherence to physical distancing. Furthermore, we also explored whether viral anxiety of healthcare workers mediated the influence of intolerance of uncertainty on adherence to physical distancing.

## Methods

### Participants and Procedure

This online survey was conducted among healthcare workers at the ASAN Medical Center, University of Ulsan College of Medicine, Seoul, Korea on November 29, 2021. ASAN Medical Center is the largest tertiary hospital in South Korea, where a total of 9,216 workers (1,759 medical doctors, 4,526 nursing professionals, and 2,931 other healthcare workers) are employed. Nearly all of them are Korean nationals. We recruited participants via an advertisement posted on the hospital's intranet, which stated the study's objective, enrollment procedure, and reward for participation. The participants completed the survey voluntarily, and a gift-coupon worth approximately five US dollars was provided as a reward for participation. The study protocol was approved by the Institutional Review Board (IRB) of the ASAN Medical Center (2021-1682), and the requirement to obtain written informed consent was waived by IRB. The sample size was estimated to be 300 in total, based on the calculation that there would be 10 samples per cell, with a total of 10 cells. (31) The cells were derived based on two groups of jobs (nursing professionals and others) and five groups based on age (20, 30, 40, 50, and 60s). After all, a total of 330 healthcare workers participated in this study on one day. The survey form was developed according to the Checklist for Reporting Results of Internet e-Surveys (CHERRIES) guidelines (32), and the usability and technical functionality were tested by investigators (SC). We collected the participants' ages, sexes, years of employment, and marital statuses. Responses to questions related to COVID-19 such as “Have you experienced taking care of confirmed COVID-19 patients?”, “Did you experience being quarantined due to infection with COVID-19?”, “Did you experience being infected with COVID-19?”, or “Did you get vaccinated?” were gathered. Past psychiatric history was assessed with the question “Have you experienced or been treated for depression, anxiety, or insomnia?”, and current psychiatric distress was assessed with the question “Do you think you are currently depressed or anxious, or do you feel you need help to improve your mood?”.

### Measures

#### Questionnaires on Adherence to Physical Distancing, Health Beliefs, and Perceived Social Norms

##### Adherence to Physical Distancing

Adherence to physical distancing was assessed using a questionnaire (Supplementary File 1) developed by Gouin et al. (30). It consists of seven items which can be rated on 5-point Likert scale, with higher score indicating greater adherence to physical distancing. This questionnaire was originally developed in English, and we used translated Korean version of the scale in this study (Supplementary File 2). We translated the questionnaire using a back translation method. Two bilingual experts translated the English version of the scale into two Korean versions. Then, these two translated Korean versions were synthesized into one. The synthesized version was back translated into English by two other bilingual experts, which were combined into one. Experts who translated it into Korean version compared the back-translated version and the original version to check for any discrepancy in meaning.

##### Health Beliefs and Perceived Social Norms

To assess psychosocial factors influencing adherence to physical distancing, participants completed a series of items assessing health beliefs about COVID-19 as well as perceived social norms related to physical distancing. Health beliefs includes three items for perceived susceptibility of being infected, three items for perceived severity of viral infection, three items of perceived benefit of physical distancing, and four items of barriers of following physical distancing, and one item of self-efficacy. Perceived social norms subscale contains single items assessing descriptive social norms, personal injunctive norms or moral norms, and social injunctive norms. These items were originally developed by Gouin et al. (30), and we translated into Korean language with permission from the original developer, and reversely translated it into English to check accuracy.

#### Stress and Anxiety to Viral Epidemics-6 Items (SAVE-6)

The SAVE-6 scale is a self-rating scale for measuring one's viral anxiety (33), and was derived from the SAVE-9 scale for measuring healthcare workers' work-related stress and anxiety response in relation to viral epidemics (34). The SAVE-9 consists of nine items which can be clustered into two factors; the SAVE-6 labeled “anxiety about the epidemic” (items 1, 2, 3, 4, 5, and 8), and the SAVE-3 labeled “work-related stress associated with the epidemic” (items 6, 7, and 9). All nine items can be rated using a 5-point Likert scale ranging from 0 (*never*) to 4 (*always*). In this study, we used the original Korean version of the SAVE-6 scale rather than SAVE-9, because we tried to explore the effect of viral anxiety of healthcare workers on adherence to physical distancing. The Cronbach's alpha among this sample was 0.805.

#### Patient Health Questionnaire-9 Items (PHQ-9)

The PHQ-9 is a self-report questionnaire that measures severity of depression (35). It consists of nine items, rated from 0 (*not at all*) to 3 (*nearly every day*). In this study, we used the Korean version of the PHQ-9 (36). The Cronbach' alpha was.883 in this sample.

#### Intolerance of Uncertainty-12 Items (IUS-12)

The IUS-12 is a shortened version of the original IUS (37). It is a self-rating questionnaire that measures one's intolerance of uncertainty. It consists of 12 items which are rated according to the respondent's level of agreement (1 to 7). Higher total scores reflect greater intolerance of uncertainty. In this study, we applied the Korean version of the IUS-12 (38), and Cronbach's alpha among this sample was 0.842.

### Statistical Analysis

First, we explored the reliability and validity of the Korean version of the questionnaires on adherence to physical distancing, health beliefs, and perceived social norms among the healthcare worker sample. We checked the correlation matrix and determinant value to identify the multicollinearity among items. We also checked the adequacy of the matrix correlations for the Exploratory Factor Analysis (EFA) based on Kaiser-Meyer-Olkin (KMO) value and Bartlett's test of sphericity. Before running the EFA, we performed parallel analysis and scree plot to identify the number of factors to retain for subsequent rotation. In EFA, principal component analysis (PCA) was utilized. We warranted oblique rotation to assess the correlations between extracted factors. As all the correlations between factors were significant except one, we retained this oblique (oblimin) rotation method. In this study, we explore the appropriate model of questionnaire on adherence to physical distancing using seven items and health beliefs using 13 items (three items for perceived susceptibility of being infected, three items for perceived severity of viral infection, three items for perceived benefits of physical distancing, and 4 items for barriers to following physical distancing). The single self-efficacy item was excluded in this model, as single items could not be included for the factor analysis. In addition, since the three items in the social norms subcategory measure different types of social norms as a single measurement, we did not include those in the final model. The reliability (internal consistency) was examined using the Cronbach's alpha and McDonald's omega. The convergent validity was examined based on a Pearson's correlation analysis with other rating scales.

Second, we explored the association of Adherence to Physical Distancing Scale with other rating scales. Demographic characteristics and rating scales scores are summarized as mean ± standard deviation. The level of significance for the analyses were defined as two-tailed at values of *p* < 0.05. Continuous variables were analyzed using a student's t-test, and categorical variables were analyzed using a Chi-square test. A linear regression analysis was performed to reveal the predicting variables for adherence to physical distancing. The bootstrap method with 2,000 resamples was implemented to examine the mediation effect. We used SPSS version 21.0, AMOS version 27 for Windows (IBM Corp., Armonk, NY, USA), and JASP version 0.14.1 to perform the statistical analysis.

## Results

A total of 330 healthcare workers participated in this survey. All except one agreed to allow their responses to be used for the study purposes. Hence, 329 responses were analyzed after excluding the response of the worker who did not agree for their response to be used in the study (Table 1).

**Table 1:** Clinical characteristics of participants (*N* = 329).

**Variables**	**N (%) Mean ±SD**
**Sex (female)**	267 (81.4%)
**Age**	35.8 ± 14.3
**Years of employment**	9.7 ± 7.7
**Job**	
Nursing professionals	194 (59.0%)
Doctors	23 (7.0%)
Other healthcare workers	112 (34.0%)
**Marital status**	
Single	157 (47.7%)
Married, without kids	51 (15.5%)
Married, with kids	121 (36.8%)
**Are you a shift worker? (Yes)**	73 (22.3%)
**Questions on COVID-19**	
Did you experience being quarantined due to infection with COVID-19? (Yes)	45 (13.7%)
Did you experience being infected with COVID-19? (Yes)	2 (0.6%)
Did you get vaccinated? (Yes)	327 (99.4%)
**Psychiatric history**	
Did you have experience or been treated for depression, anxiety, or insomnia? (Yes)	46 (13.9%)
Do you think you are currently depressed or anxious, or do you need help to improve your mood? (Yes)	24 (7.3%)

### Study 1: Reliability and Validity of the Korean Version of Questionnaires on Adherence to Physical Distancing and Health Beliefs

The normality assumption for items in both the adherence to physical distancing and health beliefs questionnaires were checked based on the skewness and kurtosis within the range of ± 2 (Table 2). Correlation matrices shows the absence of very high correlation (≥0.90) among items of both scales. These correlations suggest lack of multicollinearity problems. Determinant values (0.0402 for the adherence to physical distancing, and 0.0002 for the health beliefs) are above the suggested cut-off (>0.00001) and support the absence of multicollinearity among items. Data suitability and sampling adequacy for factor analysis were assessed based on the KMO measure (0.820 and 0.768, respectively) and Bartlett's test of sphericity (*p* < 0.001). Parallel analysis which suggested four factors in the health beliefs and two factors in the adherence to physical distancing. Next, a scree plot and EFA with oblimin rotation advised the four factors model of health beliefs (factor I - perceived susceptibility, factor II - perceived severity, factor III - perceived benefits, and factor IV - perceived barriers), and two factors model of adherence to physical distancing [factor I - adherence to physical distancing part I (items 1, 2, 3, 4, and 5), and factor II - adherence to physical distancing part II (items 6 and 7)]. Factor loading of items in each scale are presented in Table 2. The two extracted factors of adherence to physical distancing questionnaire explained 70.8% variance (factor I explained 52.2%, and factor 2 explained 18.6% variance). The four extracted factors of health beliefs questionnaire explained 77.6% variance (factor I explained 8.2%, factor II explained 14.1%, factor III explained 24.2%, and factor IV explained 31.1% variance, Supplementary Table 1).

**Table 2:** Factor structure of the Korean version of the questionnaires on adherence to physical distancing and health beliefs (*N* = 329).

**Items**	**Response scale (%)**					**Descriptive Statistics**		**CITC**	**CID**	**Factor loading (EFA)**
	**0**	**1**	**2**	**3**	**4**	**M**	**SD**			
**Health beliefs**										
Susceptibility item 1	8.5**%**	33.4**%**	44.1**%**	13.1**%**	0.9**%**	2.64	0.85	0.747	0.817	0.851
Susceptibility item 2	3.3**%**	33.1**%**	48.0**%**	14.3**%**	1.2**%**	2.77	0.78	0.854	0.724	0.919
Susceptibility item 3	1.5**%**	21.9**%**	39.5**%**	33.4**%**	3.6**%**	3.16	0.86	0.659	0.899	0.795
Severity item 1	1.8**%**	30.1**%**	43.2**%**	21.9**%**	3.0**%**	2.94	0.84	0.779	0.834	0.945
Severity item 2	1.2**%**	22.2**%**	48.0**%**	25.2**%**	3.3**%**	3.07	0.81	0.826	0.793	0.860
Severity item 3	1.2**%**	14.9**%**	44.7**%**	33.7**%**	5.5**%**	3.27	0.83	0.725	0.881	0.769
Benefit item 1	5.8**%**	26.4**%**	30.7**%**	31.9**%**	5.2**%**	3.04	1.01	0.803	0.878	0.902
Benefit item 2	5.2**%**	19.1**%**	25.2**%**	37.4**%**	13.1**%**	3.34	1.09	0.815	0.868	0.926
Benefit item 3	6.4**%**	22.2**%**	31.0**%**	33.4**%**	7.0**%**	3.13	1.04	0.829	0.855	0.912
Barrier item 1	48.0**%**	20.7**%**	24.9**%**	6.1**%**	0.3**%**	1.90	1.00	0.341	0.886	0.555
Barrier item 2	6.7**%**	20.7**%**	31.6**%**	32.5**%**	8.5**%**	3.16	1.06	0.740	0.708	0.869
Barrier item 3	3.6**%**	17.0**%**	28.3**%**	35.0**%**	16.1**%**	3.43	1.06	0.719	0.719	0.878
Barrier item 4	7.3**%**	26.1**%**	39.5**%**	21.0**%**	6.1**%**	2.92	1.00	0.758	0.703	0.885
**Adherence to physical distancing**										
Distancing item 1	5.8**%**	11.6**%**	18.8**%**	40.7**%**	23.1**%**	3.64	1.13	0.774	0.821	0.818
Distancing item 2	3.3**%**	9.4**%**	20.4**%**	41.0**%**	25.8**%**	3.77	1.04	0.780	0.819	0.891
Distancing item 3	2.1**%**	8.5**%**	16.7**%**	35.0**%**	37.7**%**	3.98	1.04	0.711	0.835	0.838
Distancing item 4	3.6**%**	9.4**%**	11.6**%**	27.4**%**	48.0**%**	4.07	1.14	0.550	0.874	0.818
Distancing item 5	7.0**%**	11.9**%**	28.6**%**	38.3**%**	14.3**%**	3.41	1.09	0.376	0.642	0.675
Distancing item 6	0.6**%**	1.5**%**	3.3**%**	11.9**%**	82.7**%**	4.75	0.65	0.422	0.642	0.906
Distancing item 7	0.9**%**	0.9**%**	2.4**%**	9.1**%**	86.6**%**	4.80	0.61	0.774	0.821	0.902

*CID, Cronbach's alpha if item deleted; CI, confidence interval*.

The questionnaire on adherence to physical distancing showed good reliability, when we tested for all items (Cronbach's alpha = 0.844, McDonald's Omega = 0.868). Cronbach's alpha of each factor was good (0.868 for distancing factor I and 0.781 for distancing factor II). The Cronbach's alphas if the dropped items were measured were 0.703–0.899 (Table 2). The convergent validity based on the Pearson's correlation analysis is presented in Table 3. The health beliefs questionnaire also showed the good reliability for items excluding self-efficacy (Cronbach's alpha = 0.756, McDonald's Omega = 0.717). Each factor also showed a good reliability (0.868 for perceived susceptibility, 0.885 for perceived severity, 0.907 for perceived benefit, and 0.812 for perceived barrier).

**Table 3:** Correlation coefficients of each variable in all participants.

**Variables**	**Age**	**1**	**2**	**3**	**4**	**5**	**6**	**7**	**8**	**9**
**1. Adherence to physical distancing**	0.06									
**2. Perceived susceptibility**	0.02	0.09								
**3. Perceived severity**	0.002	0.21^**^	0.58^**^							
**4. Perceived benefit**	0.13^*^	0.29^**^	0.11^*^	0.13^*^						
**5. Perceived barrier**	−0.04	−0.01	0.13^*^	0.16^**^	−0.20^**^					
**6. Descriptive social norms**	0.11^*^	0.04	−0.21^**^	−0.08	0.15^**^	−0.04				
**7. Personal injunctive norms**	−0.03	0.41^**^	−0.01	0.13^*^	0.48^**^	−0.23^**^	0.21^**^			
**8. SAVE-6**	0.001	0.23^**^	0.45^**^	0.45^**^	0.11^*^	0.17^**^	−0.16^**^	0.09		
**9. PHQ-9**	−0.05	0.06	0.27^**^	0.26^**^	−0.06	0.24^**^	−0.14^**^	−0.04	0.03	
**10. IUS-12**	−0.10	0.14^*^	0.18^**^	0.13^*^	−0.07	0.15^**^	−0.11^*^	0.03	0.03	0.36^**^

*SAVE-9, Stress and Anxiety to Viral Epidemics-9 items; PHQ-9, Patient Health Questionnaire-9 items; IUS-12, Intolerance of Uncertainty-12 items*.

* *p < 0.05*,

** *p < 0.01*.

### Study 2: Viral Anxiety, Depression, Intolerance of Uncertainty, and Adherence to Physical Distancing

Table 3 shows that the adherence to physical distancing score was significantly correlated with perceived severity (r = 0.21, *p* < 0.01), perceived benefits (r = 0.29, *p* < 0.01), personal injunctive norms (r = 0.41, *p* < 0.01), SAVE-6 score (r = 0.23, *p* < 0.01), and IUS-12 score (r = 0.14, *p* < 0.05).

We used the linear regression analysis to explore which variables predicted the adherence to physical distancing among healthcare workers. The results of the analysis showed that the variables that were significantly correlated with adherence to physical distancing were perceived severity, perceived benefit, personal injunctive norms, SAVE-6, and IUS-12 scores; these were included in the final model. However, we excluded perceived severity in the final model since we believed that there could be multicollinearity issue with viral anxiety (SAVE-6). Furthermore, the results also revealed that adherence to physical distancing among healthcare workers was predicted by perceived benefits of physical distancing (β = 0.12, *p* = 0.03), personal injunctive norms (β = 0.33, *p* < 0.001), SAVE-6 score (β = 0.13, *p* = 0.01), and IUS-12 score (β = 0.11, *p* = 0.04; adjusted R^2^ = 0.20, F = 14.8, *p* < 0.001; Table 4).

**Table 4:** Linear regression analysis to explore the predicting factors for adherence to physical distancing among healthcare workers.

**Dependent variables**	**Included parameters**	**Beta**	***P*-value**
Adherence to physical	Age	0.04	0.39
distancing	Sex	−0.003	0.96
	Perceived benefit	0.12	0.03
	Personal injunctive norms	0.33	< 0.001
	SAVE-6	0.13	0.01
	IUS-12	0.11	0.04

*SAVE-6, Stress and Anxiety to Viral Epidemics - 6 items; IUS-12, Intolerance of Uncertainty Scale-12 items*.

**Figure 1:**
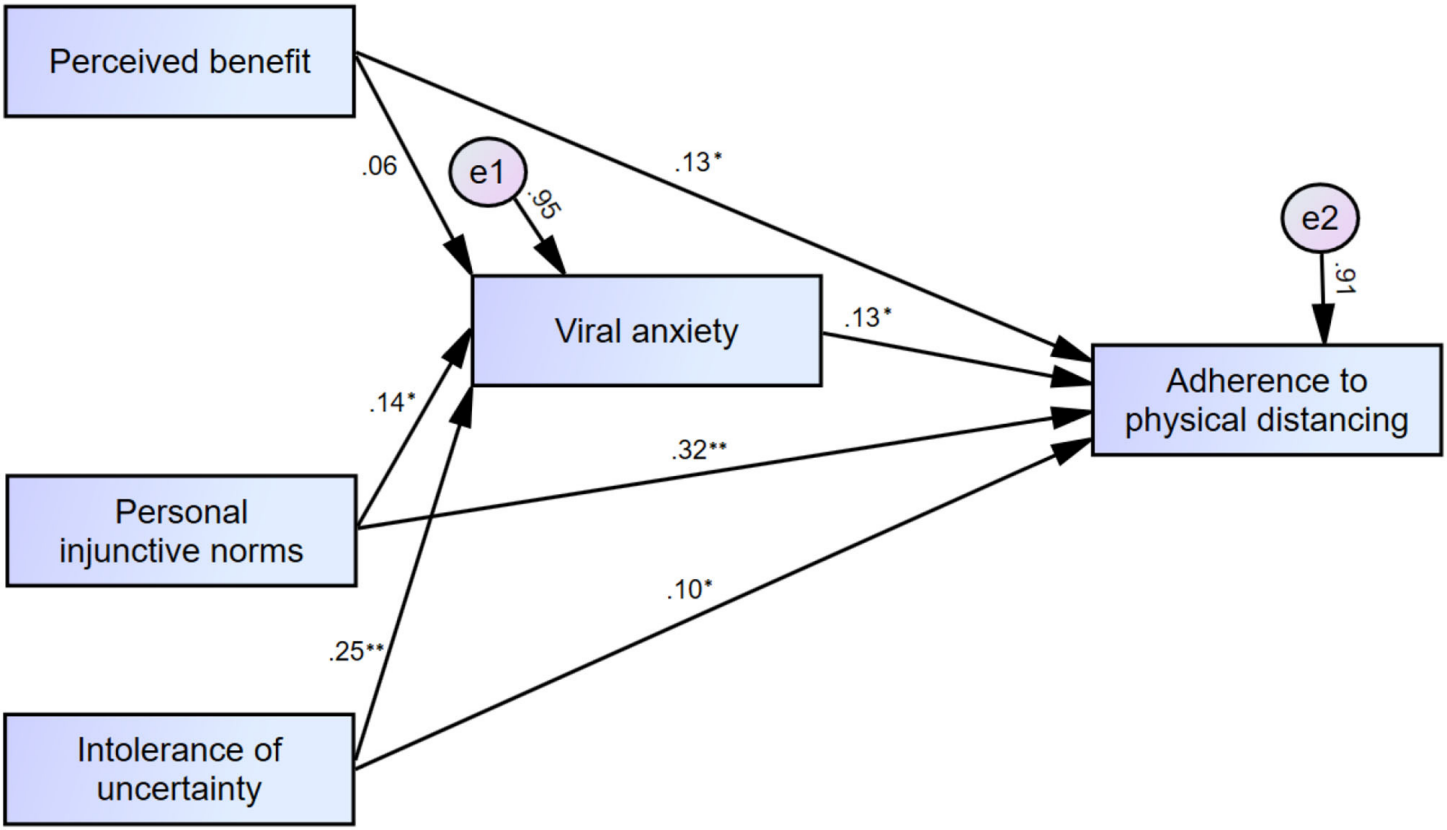
Mediation model showing the pathway from the effect of perceived benefits, personal injunctive norms, and intolerance of uncertainty (independent variables) on adherence to physical distancing (outcome) through viral anxiety (mediator). *p < 0.05, **p < 0.01.

Mediation analysis (Table 5, Figure 1) showed that perceived benefits of physical distancing, personal injunctive norms, and intolerance of uncertainty directly influenced adherence to physical distancing. The viral anxiety of healthcare workers mediated the association between intolerance of uncertainty and adherence to physical distancing but not the influence of perceived benefits and personal injunctive norms on adherence to physical distancing.

**Table 5:** The results of mediation analysis.

**Effect**	**Standardized estimate**	**S.E**.	***Z*–value**	***P***	**95% CI**
**Direct effect:**					
Perceived benefit → Adherence to physical distancing	0.13	0.10	2.33	0.02	0.04 to 0.41
Personal injunctive norms → Adherence to physical distancing	0.32	0.33	5.74	<0.01	1.28 to 2.61
IUS-12 → Adherence to physical distancing	0.10	0.05	2.03	0.04	0.003 to 0.18
**Indirect effect:**					
Perceived benefit → SAVE-6 → Adherence to physical distancing	0.01	0.01	0.98	0.33	−0.01 to 0.04
Personal injunctive norms → SAVE-6 → Adherence to physical distancing	0.02	0.06	1.74	0.08	−0.01 to 0.24
IUS-12 → SAVE-6 → Adherence to physical distancing	0.03	0.01	2.27	0.02	0.004 to 0.06
**Component**					
Perceived benefit → SAVE-6	0.06	0.09	1.06	0.29	−0.08 to 0.27
SAVE-6 → Adherence to physical distancing	0.13	0.06	2.58	0.01	0.04 to 0.27
Personal injunctive norms → SAVE-6	0.14	0.31	2.36	0.02	0.12 to 1.34
IUS-12 → SAVE-6	0.25	0.04	4.81	<0.001	0.12 to 0.28
**Total effect:**					
Perceived benefit → Adherence to physical distancing	0.14	0.10	2.45	0.01	0.05 to 0.43
Personal injunctive norms → Adherence to physical distancing	0.34	0.34	6.05	<0.001	1.39 to 2.73
IUS-12 → Adherence to physical distancing	0.14	0.05	2.75	0.006	0.04 to 0.21

*S.E., standard error; CI, confidence interval*.

*SAVE-6, Stress and Anxiety to Viral Epidemics-6 items, US-12, Intolerance of Uncertainty-12 items*.

## Discussion

In this study, first, we observed that the Korean version of questionnaires on adherence to physical distancing and health beliefs was valid and reliable. Second, adherence to physical distancing among healthcare workers was predicted by the perceived benefits of physical distancing, personal injunctive norms, viral anxiety, and intolerance of uncertainty. Adherence to physical distancing was directly influenced by its perceived benefit, personal injunctive norms, and intolerance of uncertainty. Viral anxiety of healthcare workers mediated the association between intolerance of uncertainty and adherence to physical distancing.

### Reliability and Validity of the Korean Version of the Adherence to Physical Distancing and Health Beliefs Questionnaires

In this study, we conducted a factor analysis using 13 items of health beliefs and seven items of adherence to physical distancing questionnaires, excluding the self-efficacy item in the health beliefs subcategory. Three items of perceived social norms questionnaire also were not tested. These three items are thought to reflect different types of social norms, and we would expect that they do not load on the same factors. The four factors model of health beliefs and two factors model of adherence to physical distancing questionnaires showed a good validity among healthcare workers. However, the factor loading value of item 1 of the perceived barriers to physical distancing (“How costly or expensive is the application of these recommendations for you?”) was relatively low (0.342) among this sample. In this sample, 48.0% of participants responded “not at all” to this item (Table 2) unlike the responses to other items. We can speculate that these results come from the fact that healthcare workers who are working in hospitals suffer fewer financial problems from physical distancing policy compared to other people working in other businesses or workplaces which were financially influenced by the lockdown. Another possible explanation is that healthcare workers would observe physical distancing regardless of its cost because of their sense of duty.

The reliability tests results showed that the Korean version of questionnaires on adherence to physical distancing and health beliefs can be applied to healthcare workers. It also showed good convergent validity with pre-existing rating scale for viral anxiety. Components of the questionnaires were significantly positively correlated with high level of viral anxiety (SAVE-6 score, Table 3), though some components of the scale were not significantly correlated with depression (PHQ-9 score). We speculate that high levels of viral anxiety may influence the adherence to physical distancing to prevent from the viral infection.

### Adherence to Physical Distancing, Perceived Benefits, and Personal Injunctive Norms

In this study, we observed that perceived benefits of physical distancing, personal injunctive norms, intolerance of uncertainty, and viral anxiety were associated with adherence to physical distancing. Previous studies also showed that the perceived benefits of physical distancing are a significant predictor for the adherence to physical distancing among the general population (30). Based on the health beliefs model (39), if individuals think that a negative health outcome will be severe, they can perceive the benefits of behavior which can reduce the higher likelihood of negative outcome, and the perceived benefits of behavior can predict behavior (40). Evolutionarily, collective threats will be cleared if groups make clear and strict rules to be adhered to (41). The perceived benefits of physical distancing can be enhanced by enhancing knowledge of physical distancing to reduce the spread of COVID-19. Among the general population in Australia, knowledge of the restrictions was reported to predict intention to adhere to physical distancing (42). Of course, we can consider that healthcare workers may better understand the benefits of physical distancing, and about 70% of participants were nursing professionals or medical doctors in this study. We observed that viral anxiety did not mediate the influence of perceived benefits of physical distancing on adherence to physical distancing. This may be because viral anxiety does not influence physical distancing behavior of healthcare workers who already know the benefit of physical distancing.

Injunctive norms refer to an individual's perceptions of what behaviors are acceptable or unacceptable by others, and descriptive norms refer to individuals' perceptions of which behaviors are typically performed based on observations of others (43). Injunctive norms indicate those cases in which individuals behave because they believe it is the right thing to do (unconditional preference), or because they expect others to behave and believe that others think that individuals should do so as well (conditional preference) (44).

In this study, personal injunctive norms of healthcare workers directly influence adherence to physical distancing. It was reported that personal injunctive norms were one of the strongest predictors of adherence to physical distancing (45, 46) or preventive behaviors that have consequences for the welfare of others (47). This result shows us that interventions appealing to responsibility toward society may enhance adhering to physical distancing in this pandemic. However, we also observed that viral anxiety did not mediate the influence of personal injunctive norms on adherence to physical distancing. Like the lack of mediation effect of viral anxiety on the relationship between perceived benefits from and adherence to physical distancing, it also might come from the fact that viral anxiety does not influence physical distancing behavior of healthcare workers who already were following social norms for their, their family's, and neighbors' safety.

### Adherence to Physical Distancing, Intolerance of Uncertainty, and Viral Anxiety

We observed that intolerance of uncertainty directly influenced adherence to physical distancing. Intolerance of uncertainty may be associated with the tendency to react negatively to uncertain situations. In the COVID-19 era, healthcare workers may find it difficult to tolerate the uncertainty associated with the spread of COVID-19. This may cause them to enhance their adherence to physical distancing in order to ensure the safety of their patients and themselves. In addition, viral anxiety, measured with a rating scale specific to the viral epidemic, mediated the influence of the intolerance of uncertainty on adherence to physical distancing in this study. The viral anxiety of healthcare workers might play a role in enhancing their adherence to physical distancing. Healthcare workers usually worry about transmitting the virus from hospital to their home or from outside of the hospital to inside of the hospital. Especially female nursing professionals or juniors can have higher levels of viral anxiety (2). If they have difficulty tolerating the uncertainty and they feel a higher level of viral anxiety, they will adhere to physical distancing more.

The effect of intolerance of uncertainty is complex. Among university students, intolerance of uncertainty was reported to mediate the relationship between fear of COVID-19 and procrastination (48). This shows that people escape from the risky places when they sense harm. Conversely, physical distancing can reduce one's anxiety level. In a study, perceived compliance with physical distancing was associated with lower levels of anxiety symptoms (16). Viral anxiety might also induce adherence to physical distancing, and well adapted physical distancing may reduce anxiety symptoms. To tease out the directionality of this relationship, longitudinal studies are needed.

In this study, depression was not associated with adherence to physical distancing. In the correlation analysis, depressive symptoms measured with the PHQ-9 were significantly associated with perceived susceptibility, perceived severity, perceived barriers, and viral anxiety, but adherence to physical distancing was not significantly correlated with depression. In general, depression was considered to be associated with low adherence to or compliance with medical advice (49). Based on the theme, we can expect that healthcare workers' depression could be related to lower adherence to physical distancing. There may be a few reasons for the lack of significant correlation between depression and adherence to physical distancing in this study. First, healthcare workers will adhere to physical distancing during the COVID-19 pandemic regardless of whether they feel depressed or stressed, as they believe that adhering to physical distancing is their duty or that it contributes to the safety of themselves and the patients they care for (50). Another possible explanation is that they have already adapted well to the stress or depressed mood associated with having to work continuously throughout the COVID-19 pandemic, and they adhere to physical distancing regardless of their state of depression.

There are limitations in this study. First, the responses collected via a self-report web-based questionnaire may be biased. Due to the pandemic situation, we decided to collect participants' responses via online survey rather than the face-to-face interview to prevent the transmission of the virus. Second, the survey was conducted only in one hospital located in Seoul, and it cannot be generalized to other sites. Third, we were unable to classify workers as patient-facing, contact, or frontline healthcare workers. In addition, the participants are considered to be clinically vulnerable or living with family or friends who would be considered as clinically vulnerable. This may have also influenced the results.

In conclusion, we observed that the Korean version of adherence to physical distancing and health beliefs questionnaires were applicable to healthcare workers and had good reliability and validity. In addition, we observed that adherence to physical distancing was directly influenced by the perceived benefits of physical distancing, personal injunctive norms, and intolerance of uncertainty. Viral anxiety of healthcare workers mediated the association between intolerance of uncertainty and adherence to physical distancing. In the era of the “living with coronavirus” policy in Korea, it is important to manage healthcare workers' intolerance of uncertainty and enhance their perception regarding the benefits of physical distancing to encourage better adherence to physical distancing policy, which can prevent virus transmission during the pandemic for the safety of healthcare workers and patients whom they take care of.

## Data Availability Statement

The raw data supporting the conclusions of this article will be made available by the authors, without undue reservation.

## Ethics Statement

The study protocol was approved by the Institutional Review Board (IRB) of the Asan Medical Center (2021-1682) and the requirement to obtain written informed consent was waived by IRB. Written informed consent for participation was not required for this study in accordance with the national legislation and the institutional requirements.

## Author Contributions

SC and WS: conceptualization. SC, TL, and YH: data curation. SC, OA, J-PG, and WS: formal analysis. SC and TL: investigations. SC, J-PG, and YH: methodology. SC: project administration. YH, OA, and TL: visualization. SC, TL, OA, WS, YH, and J-PG: writing—original draft. All authors: writing—review and editing. All authors contributed to the article and approved the submitted version.

## Conflict of Interest

The authors declare that the research was conducted in the absence of any commercial or financial relationships that could be construed as a potential conflict of interest.

## Publisher's Note

All claims expressed in this article are solely those of the authors and do not necessarily represent those of their affiliated organizations, or those of the publisher, the editors and the reviewers. Any product that may be evaluated in this article, or claim that may be made by its manufacturer, is not guaranteed or endorsed by the publisher.

## Footnotes

^1^Available online at: https://covid19.who.int/ (accessed on March 28, 2022).

^2^Available online at: http://ncov.mohw.go.kr/en/ (accessed on March 28, 2022).
