# Supplementary material for: Viral Anxiety Mediates the Influence of Intolerance of Uncertainty on Adherence to Physical Distancing Among Healthcare Workers in COVID-19 Pandemic
Source: Front Psychiatry. 2022 Jun 6;13:839656. doi: 10.3389/fpsyt.2022.839656 (PMC9207240; doi:10.3389/fpsyt.2022.839656)
Supplement: Supplementary file 1 [file Data_Sheet_1.PDF]

I. Health beliefs

**Health beliefs**<br><br>Thinking about the current situation related to the coronavirus COVID-19, please rate the following statements with regard to the **last two weeks**:					
**Perceived susceptibility**	**Not at all**	**Slightly**	**Moderately**	**Very**	**Extremely**
1. How susceptible do you think you are to get infected or catch the virus?					
2. How susceptible do you think your loved ones are to get infected or to catch the virus?					
3. How susceptible do you think members of your community are to get infected or to catch the virus?					
**Perceived severity**	**Not at all**	**Slightly**	**Moderately**	**Very**	**Extremely**
1. If you get infected or catch the virus, how dangerous is the virus for you?					
2. If they get infected or catch the virus, how dangerous is the virus for your loved ones?					
3. How dangerous is the virus for members of your community?					
**Perceived benefit**	**Not at all**	**Slightly**	**Moderately**	**A lot**	**Extremely**
1. How effective do you think these social distancing recommendations are in protecting you against COVID-19?					
2. To what extent do you feel that maintaining social distancing is helping others, especially older or vulnerable individuals close to you?					
3. To what extent do you feel that maintaining social distancing is helping to solve the COVID-19 crisis?					
**Perceived barrier**	**Not at all**	**Slightly**	**Moderately**	**A lot**	**Extremely**
1. How costly or expensive is the application of these recommendations for you					
2. How frustrating and unpleasant do you find the applications of these recommendations?					
3. To what extent do you feel these recommendations are becoming tiresome for you?					
4. How difficult do you find it to apply these recommendations in your daily life?					
**Self-Efficacy**	**Not at all**	**Slightly**	**Moderately**	**A lot**	**Extremely**
1. How confident are you that you will be able to follow social distancing recommendations?					

II. Adherence to physical distancing

Adherence to social distancing

In the **past two weeks**, to what extent have you been able to follow these social distancing recommendations (maintaining a physical distance with others whenever possible):

To researchers: They should now be adjusted based on current public health directives in each country

	Never	Seldom	Sometimes	Often	Almost always
1. Minimize contact with other people by staying at home					
2. Minimize non-essential travel outside of home					
3. Avoid social gatherings with several individuals at once					
4. Not having visitors in your home					
5. In public, outside of home, standing at least 2 meters away from other people					
6. Wear a face mask outside of home when a 2-meter distance cannot be maintained with others.					
7. Wear a face mask in indoor public spaces					

III. Perceived social norms

Perceived social norms

Please rate the following statements about the government directives related to social distancing (maintaining a physical distance with others whenever possible) due to COVID-19, while thinking of the **last two weeks**:

Items	Never	Slightly	Moderately	A lot	Completely
**Descriptive social norms**<br>To what extent do you think that other people in your community are following these recommendations?					
**Personal injunctive norms or moral norms**<br>To what extent do you feel that it is your civic duty to follow social distancing recommendations?					

Social injunctive norms

How do you think your close friends and family would react if they learned that you did **not** respect the social distancing recommendations (maintaining a physical distance with others whenever possible)?

⬜ They would very much disapprove of my behaviour

⬜ They would disapprove of my behaviour a little

⬜ They would neither agree nor disagree with my behaviour

⬜ They would approve of my behaviour a little

⬜ They would very much approve of my behaviour

Ref - Gouin JP, MacNeil S, Switzer A, Carrese-Chacra E, Durif F, Knäuper B. Socio-demographic, social, cognitive, and emotional correlates of adherence to physical distancing during the COVID-19 pandemic: a cross-sectional study. Can J Public Health. 2021 Feb;112(1):17-28. doi: 10.17269/s41997-020-00457-5
