# Supplementary material for: Viral Anxiety Mediates the Influence of Intolerance of Uncertainty on Adherence to Physical Distancing Among Healthcare Workers in COVID-19 Pandemic
Source: Front Psychiatry. 2022 Jun 6;13:839656. doi: 10.3389/fpsyt.2022.839656 (PMC9207240; doi:10.3389/fpsyt.2022.839656)
Supplement: Supplementary file 2 [file Data_Sheet_2.PDF]

I. 건강신념

건강신념<br><br>코로나바이러스감염증-19(이하 코로나19)와 관련된 현재 상황을 생각하면서, 지난 2주 동안에 대하여 다음 진술을 평가해 주십시오.					
지각된 민감성	전혀 아니다	약간 그렇다	보통이다	상당히 그렇다	매우 그렇다
1. 본인이 감염되거나 바이러스에 걸릴 가능성이 어느 정도라고 생각합니까?					
2. 사랑하는 사람들이 감염되거나 바이러스에 걸릴 가능성이 어느 정도라고 생각합니까?					
3. 같은 지역사회의 구성원들이 감염되거나 바이러스에 걸릴 가능성이 어느 정도라고 생각합니까?					
지각된 심각성	전혀 아니다	약간 그렇다	보통이다	상당하다	매우 그렇다
1. 본인이 감염되거나 바이러스에 걸린다면 본인에게 바이러스의 위험성이 어느 정도라고 생각합니까?					
2. 사랑하는 사람들이 감염되거나 바이러스에 걸린다면 그 사람들에게 바이러스의 위험성이 어느 정도라고 생각합니까?					
3. 같은 지역사회 구성원들에게 바이러스의 위험성이 어느 정도라고 생각합니까?					
지각된 유익성	전혀 아니다	약간 그렇다	보통이다	상당히 그렇다	매우 그렇다
1. 이러한 사회적 거리두기 권고가 코로나19로부터 본인을 보호하는 데 어느 정도 효과적이라고 생각합니까?					
2. 사회적 거리두기를 유지하는 것이 다른 사람들, 특히 가깝게 지내는 어르신이나 취약한 사람들에게 어느 정도 도움이 된다고 생각합니까?					
3. 사회적 거리두기를 유지하는 것이 코로나19 위기 해결에 어느 정도 도움이 된다고 생각합니까?					
지각된 장애성	전혀 아니다	약간 그렇다	보통이다	상당히 그렇다	매우 그렇다
1. 이러한 권고 사항을 적용하는 데 비용적으로 어느 정도로 많이 들었습니까?					
2. 이러한 권고 사항을 적용하는 것이 어느 정도로 답답하고 불쾌합니까?					
3. 이러한 권고 사항이 어느 정도로 귀찮고 피곤하게 느껴집니까?					
4. 일상생활에서 이러한 권고 사항을 적용하기가 어느 정도로 어렵습니까?					
자기효능감	전혀 아니다	약간 그렇다	보통이다	상당히 그렇다	매우 그렇다
1. 사회적 거리두기 권고를 따를 수 있다고 스스로 어느 정도로 확신합니까?					

II. 물리적 거리두기 준수

물리적 거리두기 준수

지난 2주 동안 이러한 사회적 거리두기 권고 사항(가능한 경우 다른 사람과 물리적 거리 유지)을 어느 정도 따를 수 있었는지 말씀해 주십시오.

연구자에게: 이 내용은 이제 국가별로 해당 국가의 현재 공중 보건 지침에 따라 조정해야 합니다.

항목	전혀 따르지 않았다	드물게 따랐다	가끔 따랐다	자주 따랐다	거의 항상 따랐다
1. 집에 머물면서 다른 사람과의 접촉을 최소로 줄이기					
2. 반드시 필요하지 않은 외출과 여행은 최소로 줄이기					
3. 한 번에 여러 사람이 모이는 사교 모임 피하기					
4. 집에 손님을 들이지 않기					
5. 공공장소와 집 밖에서는 다른 사람과 최소 2미터 거리두기					
6. 집 밖에서 다른 사람과 2미터 거리를 유지할 수 없는 경우 마스크 착용하기					
7. 실내 공공장소에서 마스크 착용하기					

III. 지각된 사회규범

지각된 사회규범

지난 2주를 돌아보면서, 코로나19로 인한 사회적 거리두기(가능한 경우 다른 사람과 물리적 거리 유지)와 관련된 정부 지침에 대하여 다음 진술을 평가해 주십시오.

항목	전혀 아니다	약간 그렇다	보통이다	상당히 그렇다	전적으로 그렇다
기술적 사회규범<br>지역사회의 다른 구성원들이 이러한 권고 사항을 어느 정도 따르고 있다고 생각합니까?					
개인 명령적 규범 또는 도덕규범<br>사회적 거리두기 권고를 따르는 것이 시민의 의무라고 어느 정도로 생각합니까?					

사회 명령적 규범

본인이 사회적 거리두기 권고 사항(가능한 경우 다른 사람과 물리적 거리 유지)을 존중하지 않는다는 사실을 가까운 친구와 가족이 알게 되면 어떻게 반응할 것 같습니까?

⬜ 내 행동을 매우 싫어할 것이다.

⬜ 내 행동을 조금 싫어할 것이다.

⬜ 내 행동에 동의하지도 이의를 제기하지도 않을 것이다.

⬜ 내 행동을 조금 좋아할 것이다.

⬜ 내 행동을 매우 좋아할 것이다.

참고문헌 - **Gouin JP, MacNeil S, Switzer A, Carrese-Chacra E, Durif F, Knäuper B. Socio-demographic, social, cognitive, and emotional correlates of adherence to physical distancing during the COVID-19 pandemic: a cross-sectional study. Can J Public Health. 2021 Feb;112(1):17-28. doi: 10.17269/s41997-020-00457-5**

한국어 **Seockhoon Chung, Taeyeup Lee / Dept. Psychiatry, ASAN Medical Center, Univ of Ulsan College of Medicine, Korea.** **DEC. 07. 2021**
