# Supplementary material for: Viral Anxiety Mediates the Influence of Intolerance of Uncertainty on Adherence to Physical Distancing Among Healthcare Workers in COVID-19 Pandemic
Source: Front Psychiatry. 2022 Jun 6;13:839656. doi: 10.3389/fpsyt.2022.839656 (PMC9207240; doi:10.3389/fpsyt.2022.839656)
Supplement: Supplementary file 3 [file Table_1.docx]

**Supplementary Table 1. Exploratory factor analysis outputs and reliability information of the Korean version of the Health Belief Model Scale and Adherence to Physical Distancing Scale**

| **Psychometric properties** | **Health beliefs** | | **Adherence to physical distancing** | | **Suggested cut off** |
| --- | --- | --- | --- | --- | --- |
| **Determinant** | .0002 | | .0402 | | >.00001 |
| **KMO measure** | .768 | | .820 | | .50 |
| **Bartlett’s test of sphericity** | 2691.735 (<.001) | | 1044.310 (<.001) | | Significant |
| **% Variance** | Perceived susceptibility | 8.2 | Distancing facotr_1 | 52.2 |  |
|  | Perceived severity | 14.1 | Distancing factor_2 | 18.6 |  |
|  | Perceived benefit | 24.2 |  |  |  |
|  | Perceived barrier | 31.1 |  |  |  |
| **Cronbach’s alpha** | Perceived susceptibility | .868 | Distancing facotr_1 | .868 | ≥.7 |
|  | Perceived severity | .885 | Distancing factor_2 | .781 |  |
|  | Perceived benefit | .907 |  |  |  |
|  | Perceived barrier | .812 |  |  |  |
